# The Role of Nanomedicine in Benign Gynecologic Disorders

**DOI:** 10.3390/molecules29092095

**Published:** 2024-05-01

**Authors:** Bethlehem A. Lulseged, Malini S. Ramaiyer, Rachel Michel, Eslam E. Saad, Bulent Ozpolat, Mostafa A. Borahay

**Affiliations:** 1 School of Medicine, Johns Hopkins University, Baltimore, MD 21205, USA; blulseg1@jh.edu (B.A.L.); mramaiy1@jhmi.edu (M.S.R.); 2 Department of Population, Family, and Reproductive Health, Bloomberg School of Public Health, Johns Hopkins University, Baltimore, MD 21205, USA; rmiche10@jhmi.edu; 3 Department of Gynecology and Obstetrics, Johns Hopkins University, 720 Rutland Ave, Baltimore, MD 21205, USA; esaad2@jh.edu; 4 Department of Nanomedicine, Houston Methodist Research Institute, Houston, TX 77030, USA; bozpolat@houstonmethodist.org

**Keywords:** nanomedicine, nanoparticles, uterine leiomyoma, endometriosis, PCOS, menopause

## Abstract

Nanomedicine has revolutionized drug delivery in the last two decades. Nanoparticles appear to be a promising drug delivery platform in the treatment of various gynecological disorders including uterine leiomyoma, endometriosis, polycystic ovarian syndrome (PCOS), and menopause. Nanoparticles are tiny (mean size < 1000 nm), biodegradable, biocompatible, non-toxic, safe, and relatively inexpensive materials commonly used in imaging and the drug delivery of various therapeutics, such as chemotherapeutics, small molecule inhibitors, immune mediators, protein peptides and non-coding RNA. We performed a literature review of published studies to examine the role of nanoparticles in treating uterine leiomyoma, endometriosis, PCOS, and menopause. In uterine leiomyoma, nanoparticles containing 2-methoxyestradiole and simvastatin, promising uterine fibroid treatments, have been effective in significantly inhibiting tumor growth compared to controls in in vivo mouse models with patient-derived leiomyoma xenografts. Nanoparticles have also shown efficacy in delivering magnetic hyperthermia to ablate endometriotic tissue. Moreover, nanoparticles can be used to deliver hormones and have shown efficacy as a mechanism for transdermal hormone replacement therapy in individuals with menopause. In this review, we aim to summarize research findings and report the efficacy of nanoparticles and nanotherapeutics in the treatment of various benign gynecologic conditions.

## 1. Introduction

Since the 1970s, there has been a growing interest in utilizing nanoparticles, tiny structures with unique compositions ranging from 1–1000 nanometers (nm), in therapeutics to aid in drug delivery and the treatment of various diseases [1]. Nanoparticles can be generated using lipids (i.e., micelles, liposomes, and lipid nanoparticles), polymers (i.e., Chitosan, Albumin, Poly Lactic-co-Glycolic Acid (PLGA)) and metals (i.e., iron, gold, and silver) [2,3]. They have improved water solubility, stability, pharmacokinetics, bioavailability, targetability, and safety compared to traditional drugs [4,5].

Nanoparticles are particularly useful in cancer treatment due to their excellent ability to specifically target cancer cells, penetrate deep within a tumor, avoid drug resistance, and minimize toxicities to the surrounding healthy cells [6]. In 1995, the Food and Drug Administration (FDA) approved the first nano-drug, Doxil—a polyethylene glycol coated (PEGylated) form of doxorubicin, a topoisomerase II inhibitor [7]. This drug improved doxorubicin delivery in leukemia, lymphoma, breast, uterine, ovarian, and lung cancer treatment [7]. In 2015, the FDA approved Onivyde, a PEGylated form of irinotacan, a DNA topoisomerase I inhibitor [8]. Onivyde is used to treat metastatic pancreatic cancer, and has also shown promise in treating lung cancer, glioblastomas, and other solid tumors [8].

**Figure 1:**
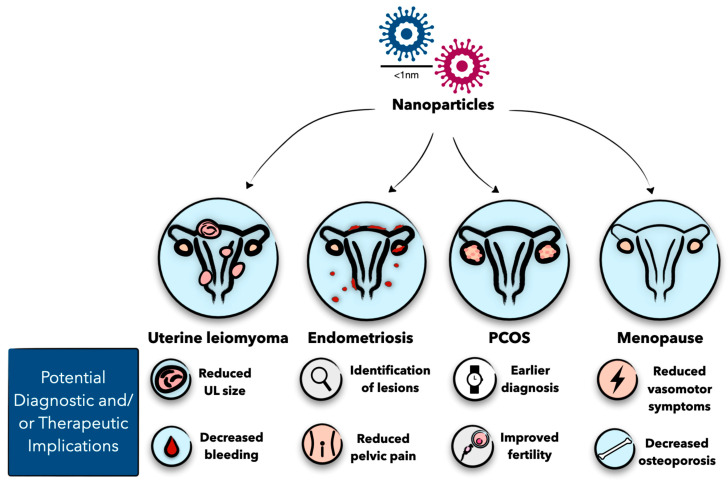
Nanoparticles can be used for the diagnosis and treatment of uterine leiomyoma, endometriosis, PCOS, and menopause. Studies have shown nanoparticles improve diagnostic options and/or patient symptoms for these benign gynecologic diseases. Figure created with Keynote.

Though nanoparticles have mostly been studied to treat cancer, the recent literature reveals they have efficacy in the treatment of other diseases. Nanomedicine-based approaches have also been applied to benign gynecologic disorders such as uterine leiomyoma, endometriosis, polycystic ovarian syndrome (PCOS), and menopause [9,10,11,12] (Figure 1). Although there are established treatment algorithms for these gynecologic conditions, there is still a need for more innovative treatment options to improve the quality of life of patients. Nanoparticles show promise in overcoming some of the current limitations related to diagnosis and drug delivery in various gynecologic conditions. However, more research is needed to understand the potential toxicities and impact on the immune system before adopting the use of nanoparticles more widely. Though there are several studies applying nanomedicine to benign gynecologic disorders, these studies have not been synthesized into one collection. In this review, we strive to demonstrate the various ways nanoparticles have been effective in the diagnosis and/or treatment of uterine leiomyoma, endometriosis, PCOS, and menopause.

## 2. Nanoparticles Overview

Nanoparticles are composed of either lipids, polymers, or metals [2,3]. Lipid-based nanoparticles (LBNPs) include liposomes, solid lipid nanoparticles (SLNs), and nanostructures lipid carriers (NLCs) [13]. LBNPs can transport hydrophobic and hydrophilic molecules and drugs while increasing drug activity and maintaining a relatively low toxicity profile, though more research is needed to understand the full scope of potential toxicities [13]. Many chemotherapy drugs have been administered using lipid-based nanoparticles, improving the efficacy of antitumor drugs [13]. Beyond their application in cancer treatment, LBNPs have also shown efficacy in delivering drugs to the lymphatic system, brain, lungs, and skin [14]. Polymeric nanoparticles (PNPs) are composed of natural polymers (i.e., gelatin, alginate, albumin) or synthetic polymers (i.e., block copolymer, grafter polymer, and ionic polymers) [15]. PNPs can be formed as nanocapsules which are composed of a polymeric shell and a hydrophobic core that hold drugs or nanospheres which are composed of a continuous polymeric network where drugs are either inside or absorbed into the PNP [16]. Ligands can be added to polymeric shells to improve drug targeting by binding the PNPs to specific receptors [17]. Due to their structure, PNPs have high mechanical strength, optical and thermal properties, and conductivity, which allow them to stabilize active therapeutic drugs and enhance delivery [15,17]. PLGA-nanoparticles are a popular form of PNPs because they produce lactic acid and glycolic acid when hydrolyzed, which can be easily metabolized in the body [18]. The use of PLGAs inspired the modification of nanoparticles with polyethylene glycol (PEG), a polymeric agent, which improves bioavailability and reduces the body’s immune response [18,19]. As a result, PEGylated nanoparticles have grown in popularity [19]. Metal-based nanoparticles are often composed of gold, silver, copper, iron, zinc, and silica, and they can be built into nanospheres, nanorods, nanostars, and other structures [20,21]. The intrinsic properties of metals allow for electrons to oscillate at the surface of the nanoparticles, giving them photothermal properties [20,21]. Metal-based nanoparticles can be conjugated with ligands or antibodies to improve the specificity of drug delivery [22]. Iron oxide nanoparticles, a popular form of metal-based nanoparticles, have been effectively used as contrast agents, drug-delivery vehicles, and thermal ablation therapeutics [20,21,22]. All three types of nanoparticles improve drug delivery and drug bioavailability, and studies have shown their efficacy in treating uterine leiomyoma, endometriosis, PCOS, and menopause (Table 1).

## 3. Uterine Leiomyoma

### 3.1. Brief Background on Uterine Leiomyoma

Uterine leiomyomas (ULs) are the most common benign tumor of the female reproductive tract [46]. While ULs are asymptomatic in up to 50% of women, UL can cause a variety of symptoms, including irregular bleeding, heavy menstrual bleeding, severe anemia, pelvic pressure, and pelvic pain [47,48]. In some cases, UL can compromise fertility, leading to early pregnancy loss and pregnancy complications, such as preterm labor, malpresentation, and postpartum hemorrhage [47,48]. The estimated prevalence of UL is 40 to 60% and 70 to 80% in individuals under 35 and individuals over 50 years old, respectively [47]. In the United States, the prevalence of UL varies by race, as Black individuals have an increased burden of disease compared with White individuals [47,49]. Furthermore, at diagnosis, Black individuals are younger, more likely to be nulligravid, and have a longer duration of symptoms due to UL [49]. The pathophysiology of UL is still not fully understood, but many factors including exposure to estrogen, obesity, stress, vitamin D deficiency, and genetic mutations may play a role in UL development [50].

Medical treatment of UL is guided by the symptoms an individual experiences. For those experiencing abnormal uterine bleeding, treatment includes a levonorgestrel intrauterine device, gonadotropin-releasing hormone (GnRH) agonists and antagonists, selective progesterone receptor modulators (SPRMs), and oral contraceptive pills (OCPs) [46,51,52]. GnRH agonists, antagonists and SPRM are also used for those with UL experiencing “bulk symptoms”, described as pelvic pressure, fullness, and heaviness [46,52]. GnRH agonists are very effective in providing symptom relief and reducing UL volume by suppressing estrogen and progesterone levels, which are important drivers of UL growth [53,54]. However, GnRH agonists are recommended for a maximum of six months to avoid negative side effects, including the loss of bone density and worsening of diabetes [55]. Therefore, GnRH agonists are commonly used in the pre-operative setting to reduce UL volume and optimize surgical removal. GnRH antagonists Elagolix and Relugolix were approved by the FDA in 2020 and 2021, respectively, and are a new orally available treatment option for UL symptom management [56]. These drugs can be used for up to 24 months, which is considerably longer than GnRH agonists; however, they are expensive and may not be easily accessible to all patients [56].

The most effective surgical treatment option for UL is hysterectomy, which is considered definitive and curative, as the uterine tissue is entirely excised [57]. However, this option is less desirable for many individuals who desire future pregnancy or want to avoid a surgery [57]. Myomectomy is another surgical option for individuals who want to keep their uterus and/or desire to preserve fertility [58]. Depending on the location of the UL, myomectomy can be performed hysteroscopically or abdominally (laparoscopy or laparotomy) for the optimal removal of UL [58].

Alternative non-surgical procedures can also be performed for those who want to avoid surgery and keep their uterus. Uterine artery embolization (UAE) is a procedure in which an embolic agent, such as polyvinyl alcohol, is injected into the uterine artery in order to block the blood supply to the uterus [59,60]. This procedure is considered safe and effective in preserving the uterus; however, the ability to preserve fertility and carry a pregnancy to term following UAE remains unclear [61]. Additionally, UL radiofrequency ablation is another non-surgical technique to reduce the UL size and bleeding associated with UL without removing the uterus. Similar to UAE, the impact on fertility following UL radiofrequency ablation remains unclear [62]. Despite the array of treatment options, medical management is not always effective in significantly improving patient symptoms and more invasive surgical measures may not be preferred by patients due to their impact on future pregnancies. Novel treatment options for UL are needed.

### 3.2. Nanomedicine for Uterine Leiomyoma

As described, the medical management of UL remains incomplete, as GnRH agonists can only be used for a maximum of six months. Many individuals do not prefer surgical interventions due to the potential threat posed to fertility. As such, recent research has investigated the efficacy of various medications delivered through nanoparticles to reduce UL size and burden. Current research remains in the pre-clinical stages, yet findings are promising for forward momentum in UL therapeutics.

#### 3.2.1. Liposomal 2-Methoxyestradiol (2-ME) Therapy

2-ME is an endogenous metabolite of estradiol, known for its pro-apoptotic nature in cancer cells, including breast cancer, prostate cancer, and UL [13,23,63]. Previous studies have found that 2-ME, an anti-tumor agent, inhibits cell proliferation and collagen biosynthesis in human and rat UL cell lines [64,65]. However, the use of 2-ME as a therapeutic agent has been hindered by low solubility and bioavailability [9]. Recent research has focused on the use of 2-ME nanoparticles in patient-derived UL tissue xenografts [9,23]. Our laboratory found that the intraperitoneal injection of liposomal 2-ME nanoparticle treatment is associated with significant tumor growth inhibition, in addition to the reduced expression of Ki67, a proliferation marker [23]. Another study evaluated the intraperitoneal injection of PEGylated poly(lactide-co-glycolide) (PEG-PLGA) nanoparticles loaded with 2-ME in UL-xenografted mice [9]. In vivo administration found 51% growth inhibition after the treatment of 2-ME-loaded nanoparticles compared to the controls [9]. These findings are a promising first step in establishing the efficacy of nanoparticles in ensuring the appropriate delivery of 2-ME to targeted tissue, though further investigation is needed to establish safety in humans [23].

#### 3.2.2. Liposomal Simvastatin Therapy

Simvastatin is a hydroxymethylglutaryl-coenzyme A (HMG-CoA) reductase inhibitor used to treat hypercholesterolemia. In UL, simvastatin has been shown to induce calcium-dependent apoptosis, decrease proliferation, and reduce extracellular matrix deposition [66,67,68]. Simvastatin, similar to 2-ME, has a low bioavailability and short half-life and requires a vector to be delivered to target UL tissue. A study by El Sabeh et al. compared treatment with simvastatin-loaded liposomal nanoparticles, subcutaneous simvastatin, and no treatment in mice xenografted with UL tissue [24]. Treatment with simvastatin-loaded liposomal nanoparticles significantly reduced UL volume and Ki67 expression in xenografted mice compared to no treatment controls [24]. Simvastatin-loaded liposomal nanoparticles did not demonstrate better outcomes compared to subcutaneous simvastatin; however, these findings did establish the feasibility of delivering simvastatin with nanoparticles [24].

#### 3.2.3. Suicide Gene Therapy

Suicide gene therapy (SGT) is defined as the integration of nucleic acids that promote apoptosis in targeted cells [25]. SGT is among the new therapeutic strategies being proposed for UL. Specifically, the Herpes Simplex Virus—Thymidine Kinase/Gancyclovir (HSV-TK/GCV) system is considered promising as it integrates into DNA and halts DNA replication, leading to the apoptosis of tumor cells [25]. However, the delivery of nucleic acids to UL cells is still being formulated. Viral vectors are established in research as achieving transfection efficiency, yet these vectors are limited by toxicity and immunogenicity [25]. These limitations have prompted investigation into non-viral vectors, including cationic polymers (i.e., PEG-modified polyplexes) and peptide-based carriers.

Peptide-based vectors have shown promising results for SGT, as they have demonstrated the ability to achieve serum resistance through polyanion coating [25]. Egorova et al. used peptide-based vectors to deliver the HSV-TK gene to primary UL cells and found an increase in apoptosis gene expression in transfected UL tissue [25]. This highlights the ability of SGT to encourage apoptosis in UL if an appropriate vector is established. As such, future directors of SGT include testing peptide-based vectors of SGT in animal models xenografted with UL tissue.

## 4. Endometriosis

### 4.1. Brief Background on Endometriosis

Endometriosis is a benign gynecological disorder affecting 6–10% of women of reproductive age [69]. It is defined as the presence of endometrial cells, glands, and stroma in locations outside the uterus [69]. In endometriosis, ectopic cells are hormone responsive and can be found on the ovaries, fallopian tubes, pelvic peritoneum and rectovaginal septum, sigmoid colon, appendix, and upper abdomen [69,70]. Patients with endometriosis may be asymptomatic, but commonly have symptoms such as dysmenorrhea, dyspareunia, dysuria, chronic pelvic pain, irregular uterine bleeding, and infertility [69]. Endometriosis is considered a multifactorial disease. While pathophysiology is not completely understood, retrograde menstruation, coelomic metaplasia, and lymphatic and vascular metastasis are thought to play a role [71]. Currently, endometriosis can be identified clinically, laparoscopically, or by imaging (MRI or ultrasound). However, MRI and ultrasound are limited in their diagnostic ability, so definitive diagnoses must be performed through diagnostic laparoscopy to visualize ectopic lesions [71,72]. Medical treatment of endometriosis is limited to nonsteroidal anti-inflammatory drugs or hormonal medications such as OCPs. The surgical excision of endometriosis lesions or removal of the entire uterus, fallopian tubes, and/or ovaries are also treatment options, though they are more invasive [71]. Given the dearth of treatment options for patients with endometriosis, there is a significant need for novel treatments.

### 4.2. Nanotherapeutics for Endometriosis Diagnosis

Laparoscopy is currently the only method to definitively diagnose endometriosis. Laparoscopy is an invasive procedure and though it is relatively safe, it comes with common surgical risks such as bleeding, infection, and damage to surrounding structures. It can also be difficult to diagnose endometriosis, even during the laparoscopy because lesions can vary in appearance, ranging from dark pigmented to pale lesions which can be hard to identify by eye [72]. For patients willing to undergo laparoscopy, nanotechnology has shown promise in improving a surgeon’s ability to identify ectopic endometrial lesions through fluorescent tagging [72]. Silicon naphthalocyanine-loaded poly(ethylene glycol)-block-poly(ε-caprolactone) (PEG-PCL) nanoparticles have successfully been used as a targeting fluorescent molecule to identify endometriosis in surgery in mouse models [26]. Additionally, after the oral administration of 5-aminolevulinic acid-induced protoporphyrin IX (5-ALA-induced PPIX), there is a preferential accumulation of 5-ALA-induced PPIX in non-pigmented endometrial lesions in patients with endometriosis, improving the identification of these lesions [73]. However, pigmented endometriosis lesions do not fluoresce after 5-ALA-induced PPIX administration. Studies show nano-delivery mechanisms can be used to improve the delivery of 5-ALA-induced PPIX to skin and gastrointestinal cancer lesions [74]. Perhaps, nanotechnology can be used to improve 5-ALA-induced PPIX delivery to pigmented endometriosis lesions. Incorporating nanoparticles to improve the fluorescence of endometriosis lesions with standard laparoscopy protocols could help surgeons identify tissue that may have been missed previously when identifying endometriosis by eye.

Many patients may not prefer to undergo invasive laparoscopic diagnostic procedures to identify endometriosis. More effective and less invasive diagnostic measures are needed. Nanotechnology in conjunction with varying imaging modalities has shown promise as an alternative to laparoscopy for endometriosis diagnosis [75]. In rat models, the intravenous injection of ultrasmall super-magnetic iron oxide (USPIO) and synthesized hyaluronic-acid-modified iron oxide nanoparticles (HAIONPs) as MRI contrast agents amplify the signal of ectopic tissue and improve the identification of endometriosis via MRI imaging [27,28,72]. In mouse models, iron oxide-based magnetic nanoparticles encapsulated in PEG-PCL-based nanocarriers successfully target endometriosis tissue by binding vascular endothelial growth factor (VEGF) receptors and increasing T2-weighted signaling via MRI [72]. NaGdF4@PEG@bevacizumab–Cy5.5 nanoparticles (NPBCNs) have also shown promise targeting VEGF in endometriotic lesions, allowing for better visualization through MRI and fluorescence imaging [29]. Nanoparticles also improve the effectiveness of photoacoustic imaging to aid in the detection of deep tissues [30]. Gold nanoparticles conjugated with a fluorescein isothiocyanate dye have shown efficacy in identifying endometriosis lesions through amplified photoacoustic signaling in mouse models [30].

### 4.3. Nanomedicine for Endometriosis Treatment

Beyond identification, nanomedicine can be used to treat endometriosis. Endometriosis is angiogenesis dependent, and factors including VEGF, bradykinin, reactive oxygen species, nitric oxide, and prostaglandins are important to the vascular permeability associated with endometriosis [72]. Nanoparticle-mediated magnetic hyperthermia is a successful cancer intervention that has been applied to endometriosis by delivering increased temperatures through heat-sensitive materials to induce the apoptosis and necrosis of target tissue [10]. Park et al. designed iron oxide-based magnetic nanoparticles encapsulated into PEG-PCL-based nanocarriers that targeted VEGF 2, a protein overexpressed in endometriotic cells, in mouse models [10]. This nanoparticle-based therapeutic was injected in mice and successfully targeted endometriosis cells, distributed throughout the entire endometriosis lesion, and increased their temperature to >46 °C in the endometriosis lesions, destroying the lesions while causing little damage to surrounding structures [10]. Though these nanoparticle-based therapeutics are promising, the intensity of the light required for treatment may limit these therapies to only be adjunct to surgical interventions [34]. VEGF has also been targeted by nanoparticles through the delivery of small interfering RNA (siRNA) to endometrial lesions in vitro and in vivo using RGD1-R6 nanoparticle carriers, reducing the size of endometriosis lesion by decreasing VEGFA expression and increasing anti-angiogenic effects [31]. Free radicals are also thought to play an important role in the pathogenesis of endometriosis [32]. In mouse models, cerium oxide nanoparticles (nanoceria) have unique anti-inflammatory properties like antioxidants, and they have shown effectiveness in decreasing oxidative stress, or damage due to increased free radicals, and angiogenesis in endometrial lesions [32]. Moreover, a new pilot study conducted in human subjects showed that methotrexate, an antiproliferative and immunosuppressive drug, carried in lipid nanoparticles improved endometriosis symptoms in study participants, though lesion size did not change [33].

Nanomedicine focused on recruiting neutrophils to target endometriosis lesions may be another treatment approach [34]. In mouse models, intraperitoneally injected bovine serum albumin–glucose oxidase nanoparticles were internalized by neutrophils in vivo and produced an anti-endometriosis effect by inducing the apoptosis of ectopic lesions through activated neutrophils [34].

## 5. PCOS

### 5.1. Brief Background on PCOS

With a worldwide prevalence of 4–20%, PCOS is a common hormonal disorder affecting reproductive-aged patients [76]. Primarily a disorder of hyperandrogenism and chronic anovulation, those with PCOS may also experience acne, amenorrhea, excessive hair growth, ovarian cysts, and infertility [77,78]. PCOS is also associated with multiple comorbidities with over 50% of patients developing prediabetes or diabetes after initial PCOS diagnosis [79]. Patients with PCOS also experience increased risk of myocardial infarction, hyperlipidemia, hypertension, anxiety, depression, and endometrial cancer [79]. Those with PCOS, who can successfully conceive, experience increased rates of miscarriage, gestational diabetes, pre-eclampsia, and premature delivery [79]. Both those at risk of and with a confirmed case of PCOS may benefit from patient education, diet and lifestyle interventions, and different therapies targeting symptoms [77]. Common medical treatments include OCPs, anti-diabetes drugs, and statins [78]. As a polygenic and multifactorial syndromic disorder, obtaining a PCOS diagnosis can be difficult. The pathophysiology of PCOS involves various pathways and proteins, rendering single genetic diagnostic tests unusable [80]. There are currently three diagnostic tools used in practice for those presenting with PCOS, which are as follows: the National Institute of Child Health and Human Development/National Institutes of Health (NICHD/NIH) Criteria (1990), which includes hyperandrogenism, oligo-ovulation/anovulation, and exclusion of other related disorders; the European Society of Human Reproduction and Embryology/American Society for Reproductive Medicine (ESHREA/ASRM) Rotterdam Criteria (2003), which includes hyperandrogenism, oligo-ovulation/anovulation, and polycystic ovaries; and the Androgen Excess Society (AES) Criteria (2006), which includes hyperandrogenism, oligo-ovulation/anovulation, polycystic ovaries, and the exclusion of other related disorders [78]. These diagnostic parameters rely on the presence of a constellation of symptoms, hormone labs (ex: follicle-stimulating hormone (FSH), testosterone, dehydroepiandrosterone-sulfate (DHEA-S), 17 hydroxyprogesterone), and the imaging of ovaries [81]. Making a PCOS diagnosis can be difficult because PCOS shares similar symptoms with other endocrine disorders and PCOS symptoms can be heterogenic [82]. There is a significant need to revolutionize PCOS diagnosis and treatment.

### 5.2. Nanomedicine for PCOS Diagnosis

Early detection of PCOS is vital for preventive therapy, preserving fertility, and improving reproductive, metabolic, and cardiovascular risks in patients [83]. Current approaches for PCOS screening are expensive, limited in their sensitivity, and time consuming. Testing the levels of hormones such as follicle-stimulating hormone (FSH) or testosterone can be helpful in reaching a PCOS diagnosis [81]; however, these tests are limited by the sensitivity of current laboratory tools. Studies show that nanoparticles may improve hormone screens. The development of a novel FSH polymer film imprinted onto the NiCosO4/rGO-modified indium tin oxide nanomaterial electrode has shown promise in PCOS diagnosis [35]. This biosensor can overcome the drawbacks of traditional FSH screening by offering a dynamic detection range with a lower limit of detection, improved sensitivity, and speed of detection at a lower cost [35]. Additionally, elevated testosterone (both total and free) can be diagnostic for excess ovarian androgens in patients with PCOS; thus, it is considered an effective compound for the early detection and monitoring of PCOS manifestations [84]. However, in PCOS, sex hormone-binding globulin (SHBG) levels are often low, possibly resulting in falsely low testosterone measurements [85]. Thus, the variability of SHBG is the preferred assay for explicating bioavailable testosterone. One study using a CS/copper-NPs/Fe3O4-NPs/GrO-NPs nanocomposite in the fabrication of a SHBG immunosensor demonstrated the improved efficacy of the immunosensor’s analytic performance and detection of SHBG [36]. Therefore, the use of nanomaterials is a robust choice for the detection of SHBG for early diagnosis of PCOS as it is faster, cheaper, and more sensitive and specific than other traditional methods [86].

### 5.3. Nanomedicine for PCOS Treatment

While standard practices may be limited, there have been recent advances in nanotechnology for treating PCOS. Recent exploration of innovative therapeutic options for PCOS has included nanoparticles such as silver and selenium, lipid-based nanocarriers, and many herbal-based nanosystems, either alone or in combination with other drugs [86]. Silver nanoparticles synthesized from *Cinnamomum zeylanicum* are useful in treating inflammation and have shown efficacy in lowering inflammatory markers in rats with PCOS [37]. This finding is important because patients with PCOS have high levels of inflammatory cytokines, so reducing inflammation in patients with PCOS may reduce or relieve their symptoms. Additionally, curcumin is a phenolic compound with anti-inflammatory and antioxidant properties [87]. Curcumin has demonstrated potential in reducing hyperglycemia, hyperlipidemia, hyperandrogenism, and insulin resistance in those with PCOS [88], but it has limited use due to its solubility and poor body pH availability [88]. However, the bio-compatible nano-curcumin has increased polarity, improved oral absorption, amplified bioavailability, and increased potential for bodily absorption, which could only further improve the symptoms associated with PCOS [86]. In fact, Raja et al. found success fabricating nanoparticles containing curcumin-encapsulated arginine and *N*-acetyl histidine-modified chitosan (CS) nanoparticles, which showed increased cellular uptake in in vitro PCOS models [11]. CS-nanoparticles are cationic polysaccharides and are widely used for the encapsulation of molecules such as antimicrobials, painkillers, and anti-inflammatory drugs [89,90]. The administration of the curcumin-encapsulated arginine and *N*-acetyl histidine-modified CS nanoparticles in rats with PCOS caused the suppression of the serum luteinizing hormone (LH), prolactin, testosterone, and insulin compared to control models [11]. This study is a promising first step in the prospective use of nanoparticles as an effective delivery platform for curcumin to treat PCOS.

Metformin is a drug commonly used for PCOS treatment, and has demonstrated positive effects of restoring ovulation, reducing weight gain, reducing androgen level circulation, reducing the risk of miscarriage, and reducing the risk of diabetes mellitus [91,92]. However, Metformin is associated with a myriad of gastrointestinal side effects including nausea, diarrhea, bloating, metallic taste, and abdominal pain [91]. Side effects are often exacerbated by repeated applications of high doses of Metformin, necessitated by its low oral bioavailability and short biological half-life [93]. Several studies have investigated metformin-containing nanoparticles and highlighted the use of nanoparticles as a drug-delivery strategy to improve its bioavailability [93]. While Metformin-containing nanoparticles have not yet been studied in the context of PCOS, researchers hypothesize that the use of nanoparticles could increase the efficacy of Metformin as a treatment for PCOS to reduce weight and improve ovulation [93].

## 6. Menopause

### 6.1. Brief Background on Menopause

Menopause describes the natural and permanent end of menstruation caused by an estrogen deficiency that is unrelated to a pathologic condition [94]. In the US, over one million individuals go through menopause annually [95]. Most previously menstruating individuals go through menopause between 45 and 56 years old; in the US, 51 is the typical age at which menopause naturally occurs [96]. Most individuals who go through menopause experience vasomotor symptoms (e.g., hot flashes and night sweats), but many other organ systems, such as the urogenital, psychogenic, and cardiovascular, can also be affected [97]. The pathophysiology of menopause is related to the reduction of ovarian follicles with age due to ovulation [94]. The levels of the anti-Mullerian hormone (AMH), inhibin B, and estradiol decrease, while LH and FSH synthesis increase [94].

Black and Hispanic individuals are more likely than White individuals to go through early and premature menopause [98]. Additionally, Black individuals frequently encounter more bothersome and longer-lasting vasomotor symptoms compared to other races [98]. Eighty percent of Black individuals who undergo menopause have vasomotor symptoms which last, on average, 10.1 years, while 65% of White individuals who undergo menopause report having vasomotor symptoms which last, on average, 6.5 years [98].

### 6.2. Nanomedicine for Menopause Treatment

Menopause has historically been treated with systemic hormonal treatment, local estrogen treatment, and selective estrogen receptor modulators to manage vasomotor and other associated symptoms. Though these treatments provide adequate symptomatic relief to many patients, the delivery of these medications to target tissues can be improved upon.

#### 6.2.1. Systemic and Local Hormonal Treatment

Systemic hormonal treatments are used in individuals undergoing menopause to treat vasomotor symptoms and insomnia, prevent osteoporosis and associated fractures, and improve cognitive function [99]. Patients can be prescribed progestin-only, estrogen-only, an estrogen–progestin combo, and estrogen–bazedoxifene for symptoms management [100]. Systemic hormone therapy should only be used for a short period of time and at its lowest effective dose because it increases the relative risk of deep vein thrombosis, stroke, and breast cancer [101].

Though hormonal therapies have shown efficacy in the treatment of a variety of menopause-related symptoms, the hormonal treatments have limited bioavailability. Studies show nanoparticles can improve the delivery and availability of systemic hormonal treatments through various delivery techniques. In a multicenter, randomized, double-blind, placebo-controlled study, Simon et al. found that patients with severe hot flashes (more than seven times/day) had a significant reduction in the frequency of moderate and severe vasomotor symptoms after receiving a micellar nanoparticle estradiol emulsion (MNPEE), compared to patients who received estradiol delivered in a placebo emulsion, likely due to improved bioavailability [38]. Botelho et al. showed the safety and efficacy of nanostructured transdermal hormone replacement therapy (10% progesterone, 0.1% estriol, and 0.25 estradiol + Biolipid/B2 nanoparticle formulations) [12]. Traditionally, transdermal menopause hormone replacement therapy is associated with an increased risk of venous thromboembolism [102]. However, patients who received nanostructured transdermal hormone replacement therapy saw an improvement in menopause symptoms with no negative health impacts after a 60-month course of medication [12]. Moreover, the lack of estrogen in menopause can increase the risk for osteoporosis due to increased bone turnover [103]. Current hormone treatments can lower the risk of osteoporosis, but Chen et al. showed that the use of a 17β-estradiol (E2)-laden mesoporous silica-coated upconversion nanoparticle with a surface modification of ethylenediaminetetraacetic acid (EDTA) in in vivo and in vitro models offered long-lasting drug release, the reversal of estrogen-deficient induced osteoporosis and the decreased damage of estrogen to the uterus [39]. Compared to the ovariectomized group, the bone mineral density in the nanocomposite treatment group was almost twice as high [39]. Nanoparticles may also aid in the delivery of hormones to improve cognition in individuals undergoing menopause. In in vitro rat models, E2-loaded Poly(lactic-co-glycolic Acid) (PLGA) nanoparticles improved learning and memory compared to E2 loaded with a vehicle control [40]. Additionally, older studies have shown the efficacy and safety of intranasally administered hormone replacement therapy, especially in postmenopausal patients with an intact uterus [104]. However, intranasal hormone replacement therapy has not been popularized. In the diseases of the central nervous system, intranasal polymeric and lipid-based nanoparticles have shown efficacy and promise in drug delivery [105]. Perhaps, nanoparticles can be valuable in improving the delivery and efficacy of intranasal hormone replacement therapy in post-menopausal patients in the future.

The genitourinary syndrome of menopause describes chronic, progressive, vulvovaginal, sexual and urinary conditions that occur during the menopausal period [106]. Patients often present with vaginal dryness and dyspareunia [106]. Symptoms can be managed with over-the-counter lubricants or with local estrogen therapy [107]. Local estrogen therapy improves vaginal atrophy by increasing blood flow, acidifying the vagina, and promoting lactobacillus dominance in the flora [108]. However, the vaginal delivery of estrogen is limited by poor adhesion and solubility [41]. Solid lipid nanoparticle (SLP)-based CS gels have shown efficacy in improving the solubility, surface area, and permeability of estrogen for vaginal delivery in in vitro models [41].

#### 6.2.2. Selective Estrogen Receptor Modulators

Selective estrogen receptor modulators (SERMs) are promising drugs for menopause management due to their ability to bind to estrogen receptors and confer agonist or antagonist effects depending on the target tissue [109]. Raloxifene (RLX) is the only SERM approved to prevent and treat osteoporosis and vertebral fractures, while preventing breast cancer in post-menopausal women [109]. Though SERMs provide many benefits, their side effects include hot flashes and venous thromboembolism [110,111]. Studies show nanoparticles are important in increasing the bioavailability of RLX. Saini et al. demonstrated that CS nanoparticles loaded with RLX had superior bioavailability, making them a potentially effective method for delivering RLX intravenously for the management of osteoporosis compared to an RLX suspension [42]. Additionally, Guo et al. showed that, when compared to an RLX suspension, RLX-loaded polymeric nanoparticles (RLX-PNPs) had significantly increased bioavailability and sustained release, causing decreased serum calcium and alkaline phosphate levels in rats with osteoporosis [43]. Additionally, the bioavailability of RLX increased with the intravenous administration of human serum albumin (HSA)-based nanoparticles (Ral/HSA/PSS NPs) or the oral administration of RLX bio-adhesive nanoparticles (RLX-bNPs) in rats [44,45]. These studies indicate that RLX-loaded nanoparticles may be a powerful nanomedicine candidate for treating postmenopausal osteoporosis at lower raloxifene dosages.

## 7. Potential Toxicity of Nanoparticles

**Figure 2:**
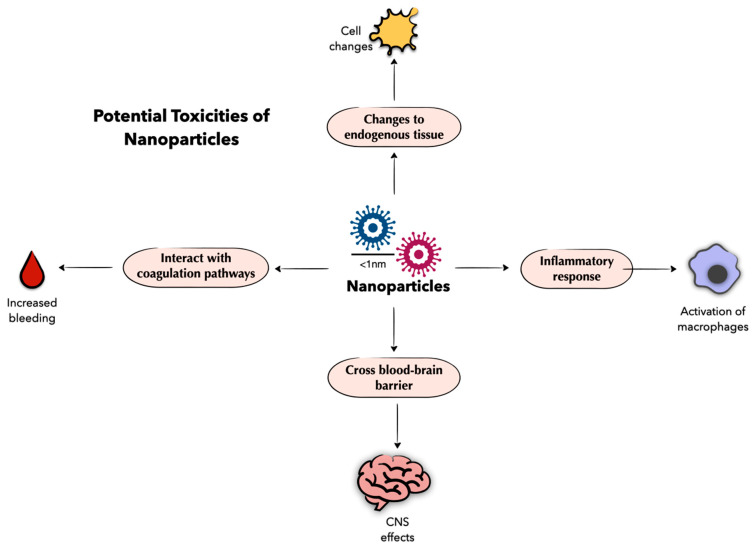
Nanoparticles are a promising therapeutic; however, they are associated with potential toxicities. Nanoparticles may cross the blood–brain barrier, interact with coagulation pathways, induce endogenous cell changes, and promote an inflammatory response, though more research is needed to determine the full extent of these and other nanoparticle-related side effects. Figure created with Keynote.

Despite the promise of nanoparticles as a treatment option for benign gynecologic disorders and other disease processes, there are limitations to the current use of nanoparticles as a primary treatment method. Nanoparticles, like any other drug, can cause toxicities at the molecular, cellular, and tissue level [112] (Figure 2). Because nanoparticles are incredibly small, they can easily move throughout the body [112]. Though studies have shown, in in vitro and in vivo models, that nanoparticles have a high binding affinity to their targets [72], the distribution and interaction of nanoparticles throughout the body—particularly across the blood–brain barrier or with the coagulation pathways—are not fully understood [113]. Nanoparticles crossing through the blood–brain barrier or interacting with the body’s ability to respond to bleeding could have potentially life-threatening effects and should be studied further. Nanoparticles may also induce endogenous tissues and cells to undergo changes that lead to significant toxicities [114]. Poland et al. showed that carbon-based nanoparticles may induce mesothelioma, a relatively rare lung cancer normally caused by asbestos inhalation in in vitro and in vivo models [115]. Additionally, Wyss et al. and Steiner et al. showed that 5-ALA-induced PPIX, a nanoparticle used to fluoresce endometriosis ectopic lesions, caused endometrial atrophy in rat and rabbit models [116,117]. There is also a risk of an immune response, or the recognition and activation of immune cells like macrophages, with nanoparticle administration. Efforts have been made to reduce immune responses by decreasing the opportunity for protein binding by coating nanoparticles with polyethylene glycol [112]. However, this coating does not completely prevent immune recognition and, in some cases, may even trigger an inflammatory response [118]. It is important to note that the side effects listed here are not unique to nanoparticles and should not necessarily prohibit their use clinically. Other drugs like chemotherapy agents have an extensive side effect profile and are still recommended for patients [119]. The specific limitation with nanoparticles is the lack of knowledge regarding the full scope of the potential toxicity of nanoparticles. Though nanoparticles are being studied in clinical studies around the world, there is limited research focused specifically on the widespread safety profile of nanoparticles [112]. More research is needed to understand the toxicities associated with each nanoparticle before they can be widely used as a standard treatment option.

## 8. Conclusions and Future Directions

In conclusion, nanoparticles are a promising new treatment option for many patients suffering from benign gynecologic conditions that lack less-invasive diagnostic and treatment options. The literature shows nanoparticles are effective in the diagnosis and/or treatment of UL, endometriosis, PCOS, and menopause in in vitro and in vivo models. However, there have been few applications of these studies in human subjects. More research is needed to understand the effectiveness and potential toxicities of using nanoparticles in patients with various benign gynecologic conditions. Nanoparticles could revolutionize the way patients are treated for UL, endometriosis, PCOS, and menopause, improving the livelihood of millions worldwide.

## Figures and Tables

**Table 1:** List of nano-therapies used for benign gynecologic disorders.

Nano-Therapy	Benign Gynecologic Disorder	Type of Nanoparticle	Original Study
Liposomal 2-ME nanoparticle	Uterine Leiomyoma	Lipid-based	[23]
PEGy-PLGA nanoparticles loaded with 2-ME	Uterine Leiomyoma	Polymeric	[9]
Simvastatin-loaded liposomal nanoparticles	Uterine Leiomyoma	Lipid-based	[24]
Peptide-based vectors for suicide gene therapy	Uterine Leiomyoma	Polymeric	[25]
Silicon naphthalocyanine loaded PEG-PCL	Endometriosis	Polymeric	[26]
Ultra-small Super-magnetic iron oxide nanoparticles	Endometriosis	Metal-based	[27]
Hyaluronic acid modified iron oxide nanoparticles	Endometriosis	Metal-based with Polymeric modification	[28]
NaGdF4@PEG@bevacizumab–Cy5.5 nanoparticles	Endometriosis	Metal-based with Polymeric modification	[29]
Gold nanoparticles conjugated with a fluorescein isothiocyanate dye	Endometriosis	Metal-based	[30]
Iron oxide-based magnetic nanoparticles encapsulated into PEG-PCL-based nanocarriers targeting VEGF 2	Endometriosis	Metal-based with Polymeric modification	[10]
SiRNA RGD1-R6 nanoparticle carriers	Endometriosis	Polymeric	[31]
Cerium oxide nanoparticles	Endometriosis	Metal-based	[32]
Methotrexate carried in lipid nanoparticles	Endometriosis	Lipid-based	[33]
Albumin-glucose oxidase-nanoparticles	Endometriosis	Lipid-based	[34]
NiCosO4/rGO modified indium tin oxide nanomaterial	PCOS	Metal-based	[35]
CS/copper-NPs/Fe3O4-NPs/GrO-NPs nanocomposite	PCOS	Metal-based	[36]
Silver nanoparticles derived from *Cinnamomum zeylanicum*	PCOS	Metal-based	[37]
Curcumin-encapsulated arginine and *N*-acetyl histidine-modified CS	PCOS	Polymeric	[11]
Micellar nanoparticle estradiol emulsion	Menopause	Polymeric	[38]
Nanostructured transdermal hormone replacement therapy	Menopause	Lipid-based	[12]
EDTA-modified 17β-estradiol (E2)-laden mesoporous silica-coated upconversion nanoparticles	Menopause	Metal-based	[39]
E2-loaded PLGA nanoparticles	Menopause	Polymeric	[40]
Solid lipid nanoparticles	Menopause	Lipid-based	[41]
CS nanoparticles loaded with RLX	Menopause	Metal-based	[42]
RLX-loaded polymeric nanoparticles	Menopause	Polymeric	[43]
Human serum albumin-based nanoparticles	Menopause	Polymeric	[44]
RLX bio adhesive nanoparticles	Menopause	Polymeric	[45]

## Data Availability

Not applicable.
